# Spatiotemporal piezoelectric microspheres for wireless endometrial repair with improved pregnancy outcomes

**DOI:** 10.1016/j.mtbio.2026.102915

**Published:** 2026-02-10

**Authors:** Rui Zhao, Shiwen Ni, Xinyu Tao, Yuxing Liu, Nengjie Yang, Chun Cheng, Yujuan Zhu, Mei Yang

**Affiliations:** a Research Center of Clinical Medicine, Affiliated Hospital of Nantong University, Medical School of Nantong University, Nantong, 226001, China; b School of Medical, Nanjing University of Chinese Medicine, Nanjing, 210023, China; c Research Center of Immunology, Affiliated Hospital of Nantong University, Medical School of Nantong University, Nantong, 226001, China

**Keywords:** Endometrial regeneration, Core-shell microspheres, Au@BTO, Polydopamine, Wireless therapy

## Abstract

Endometrial injury is a pathological damage caused by intrauterine infection, repeated miscarriage, or curettage, which can seriously impair fertility. Effective repair of damaged endometrium is crucial. Traditional interventions often only bring moderate improvements; therefore innovative repair strategies are needed. Herein, we propose a novel spatiotemporally responsive piezoelectric microsphere platform, which could be applied for ultrasound-guided endometrial regeneration and fertility recovery. These microspheres are manufactured by microfluidic technology, producing a core-shell structure: a Chinese herb-laden hydrogel embedded in piezoelectric nanoparticles, and wrapped by a viscous polydopamine (PDA) shell. The herbal hydrogel inhibits the release of inflammatory cytokines, and play the role of phytoestrogen to synergistically enhancing sexual function and tissue repair. The PDA shell scavenges reactive oxygen species (ROS), dampening oxidative stress and chronic inflammation. Meanwhile, the encapsulated gold-decorated tetragonal barium titanates (Au@BTO) nanoparticles generate bactericidal ROS upon ultrasonic activation, ensuring sterile local microenvironments. Vessel-on-a-chip and endometrium-injury rats model confirm the microspheres's outstanding compatibility, and their capacity to stimulate cell proliferation, migration and angiogenesis. Intrauterine delivery of the microspheres accelerates endometrial re-epithelialization, amplifies angiogenesis, restores myometrial integrity, and ultimately preserves fertility. With its precise spatiotemporal control and multimodal therapeutic functions, this microsphere system represents a transformative approach for localized treatment of gynecological diseases.

## Graphical abstract

**Figure:**
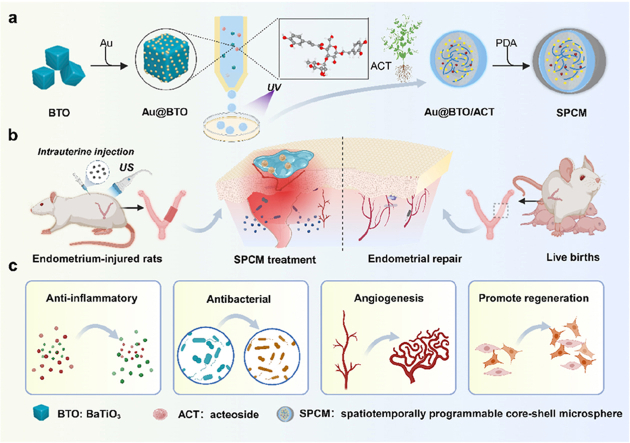

## Abbreviations

BTO: BaTiO3
UV: ultrasound
ACT: acteoside
Au@BTO: encapsulated gold-decorated tetragonal barium titanates
PDA: polydopamine
SPCM: spatiotemporally programmable core-shell microsphere
ROS: reactive oxygen species
IUAs: intrauterine adhesions
SEM: scanning electron microscopy
TEM: Transmission electron microscopy
XRD: X-ray diffraction
EDS: energy-dispersive X-ray spectroscopy
TA: Terephthalic acid
OH: hydroxyl radicals
OD: absorbance
MVNs: microvascular networks
MOUI: models of uterine injury
α-SMA: Alpha-smooth muscle actin
VEGF: Vascular endothelial growth factor

## 1 Introduction

Endometrial injury is a major threat to women's reproductive health, which can cause a range of complications, including abnormal uterine bleeding, infertility, recurrent abortion, premature birth, and birth defects [1,2]. Clinically, the main causes of endometrial injury are frequent intrauterine mechanical procedures, inflammatory infections, and drug medications [3,4]. For example, repeated miscarriage or uterine fibroid removal followed by curettage can directly damage the basal layer of the endometrium, complicating its repair process. Recently, researcher have realized the serious threat of intrauterine adhesions (IUAs) and thin endometrium to female reproductive health. Current therapies mainly include hysteroscopic adhesiolysis, intrauterine device placement, hormone therapy, stem cell-based therapy and extracellular matrix (ECM) hydrogel [[5], [6], [7], [8], [9], [10], [11]]. However, these approaches have limitations. Mechanical interventions can only alleviate uterine stenosis without restoring its original structure and function. Hormone therapy can increase endometrial thickness, but its ability to reduce fibrosis is insufficient [[12], [13], [14]]. Moreover, injected mesenchymal stem cells typically have low retention rates and the potential risk of tumorigenicity [15]. Although ECM has the advantage of tissue-specific microstructure, it belongs to a “static” natural scaffold, and its regulation has menstrual phase specificity [16]. Confronted with these challenges, there is an urgent need for a more effective therapeutic strategy [17,18].

**Fig. 1:**
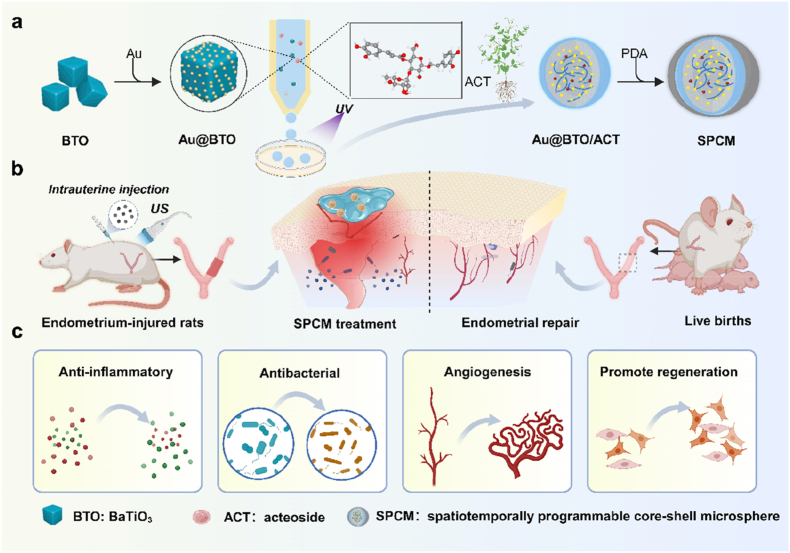
**Fabrication of spatiotemporal piezoelectric microspheres and functional mechanism for endometrial repair.** a, Overview of the synthesis process for spatiotemporal piezoelectric microspheres. b-c, In endometrium-injured rats, the present spatiotemporal piezoelectric microspheres facilitated tissue restoration. Leveraging ultrasound-enhanced transdermal delivery, the SPCM exerted anti-inflammatory, antibacterial, promoted vascular regeneration and cell proliferation. Collectively, these actions promoted healing of the damaged endometrium and markedly improved reproductive outcomes. BTO: BaTiO_3_; UV: ultrasound; ACT: acteoside; PDA: polydopamine; SPCM: spatiotemporally programmable core-shell microsphere. Schemes created with BioRender.com.

Biomaterials have become important tools for tissue repair owing to their excellent biocompatibility, shape plasticity, and functional diversity [19]. In the field of endometrial repair, functional biomaterials designed rationally can play a key role, which is expected to fundamentally compensate for the shortcomings of existing repair methods, maximize repair effects, and maintain female fertility [20]. Herein, we present an ultrasound (US)-responsive, spatiotemporally programmable core-shell microspheres (SPCM) to treat endometrium injury, as schemed in Fig. 1. The device marries two synergistic missions: (i) piezoelectrically driven tissue regeneration and (ii) on-demand immunomodulation. Piezoelectric nanomaterials have attracted increasing attention as cell stimulators (e.g. muscles, neurons) due to their ability to generate direct-current output upon mechanical deformation under various physical [21,22]. In particular, US is an effective external stimulus for piezoelectric nanomaterials owing to its easy controllability, deep tissue penetration, imaging capabilities and precise targeting to specific sites [23]. Consequently, it is believed that the combination of US with piezoelectric nanomaterials can achieve non-invasive electrical stimulation of localized tissues [24,25]. Meanwhile, hydrogel microspheres, as intelligent carriers, hold broad application prospects in biomedical fields such as cell and drug delivery, scaffold design and biofabrication, due to their high biocompatibility, strong drug-loading capacity, and spatiotemporal controllability [4,[26], [27], [28], [29]]. Integrating US effects and nano delivery systems to regulate the immune microenvironment, by leveraging the auxiliary effects of US stimulation to enhance drug delivery and material penetration, is more likely to effectively repair the injury [[30], [31], [32]]. Therefore, the design and development of multifunctional drug delivery technologies for efficient endometrial repair remain an urgent necessity.

In this study, we introduce a novel multifunctional core-shell microsphere system with spatially distributed and co-applied antagonistic materials for the wireless treatment of endometrial injury. Fabricated in a single step by microfluidic technology, each sphere harbors a Chinese-herb (acteoside, ACT) hydrogel core embedded with piezoelectric Au@BTO nanocrystals and is cloaked in an adhesive polydopamine (PDA) shell. This core-shell architecture deploys two seemingly opposing, yet complementary, redox modules: Anti-oxidative shield where the herbal matrix and PDA shell synergistically scavenge reactive oxygen species (ROS) and down-regulate inflammatory cytokines to quench chronic inflammation, Anti-bacterial core, where upon ultrasound stimulation, Au@BTO converts mechanical energy into bactericidal ROS pulses that sterilize the injured niche without systemic toxicity. In a vasculature-on-a-chip model, the microspheres displayed outstanding cytocompatibility while markedly accelerating endothelial proliferation, migration, and de novo vessel formation. Translating to a rat model of severe endometrial damage, a single intrauterine instillation reconstructed a thick, richly vascularized endometrium, restored myometrial architecture, and preserved full-term fertility. By orchestrating on-demand immunomodulation, piezoelectric stimulation, and localized drug release within one injectable platform, these microspheres set a new benchmark for spatiotemporally precise gynecologic therapies.

## 2 Results and discussion

**Fig. 2:**
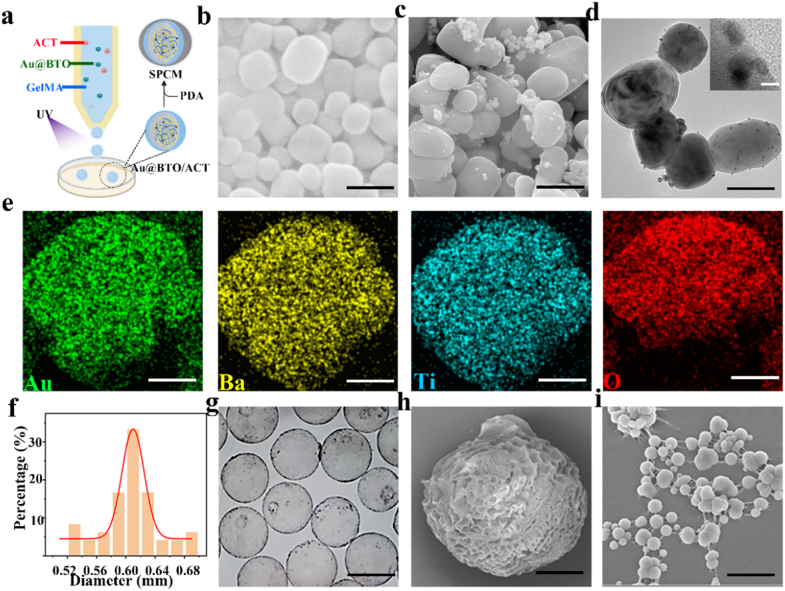
**Characterization of SPCM. a,** Schematic representation of SPCM. **b,** SEM images of BTO. Scale bars, 200 nm. **c,** SEM images of Au@BTO. Scale bars, 200 nm **d,** TEM images of Au@BTO. Scale bars, 150 nm. The upper left corner is an enlarged version. Scale bars, 5 nm. **e,** STEM-EDX elemental mapping of SPCM. **f,** Size distribution of SPCM. **g,** Bright-field image of the SPCM. Scale bars, 600 μm. **h,** SEM of the freeze-dried SPCM. Scale bar, 100 μm. **i,** SEM image of freeze-dried SPCM surface coating with PDA. Scale bars, 1 μm.

Prior to microsphere generation, Au@BTO by chemically reducing gold nanoparticles onto the surface of barium titanate nanocubes (BaTiO_3_, BTO), nanocomposites are formed. Scanning electron microscopy (SEM) imaging revealed that BTO adopts a predominantly cubic shapes (Fig. 2b), with Au NPs (∼3 nm) randomly distributed on the BTO surfaces (Fig. 2c). Transmission electron microscopy (TEM) images (Fig. 2d and insert) and selected area electron diffraction (SAED) (Fig. S2a) show crystalline lattices of the Au@BTO heterostructure. The X-ray diffraction (XRD) pattern verified the coexistence of perovskite BTO and face centered cubic gold (Fig. S2b). The shoulder peak at 39° and the splitting peak around 45° were designated as (111) and (200) planes for tetragonal phase of BTO and Au@BTO, respectively, confirming its ferroelectric/piezoelectric properties. To further verify the piezoelectric performance, we tested its ferroelectric hysteresis loops. As shown in Fig. S2c, with the increase of applied voltage, the hysteresis curve gradually opens, confirming the piezoelectric activation and providing theoretical basis and experimental support for its biological effects. To visualize the spatial distribution of Au@BTO within SPCM, energy-dispersive X-ray spectroscopy (EDS) mapping was taken. The results demonstrated that the elements for Au, Ba, Ti, and O of Au@BTO were uniformly distributed throughout the material (Fig. 2e). Then, ultrasound-responsive multicomponent piezoelectric microspheres were fabricated based on microfluidic technology. Coaxial assembly and fixation of capillaries with different inner diameters on glass slides to construct microfluidic devices. The inner capillary introduced the dispersed phase, which was a solution of Chinese herb hydrogel containing Au@BTO, while the outer capillary introduced the continuous phase, composed of liquid paraffin oil containing 5% Span (Fig. 2a). Tailoring the sheath-to-core flow-rate ratio, applied potential and microchannel dimensions enabled accurate tuning of the resulting microsphere diameter. Here, a coaxial capillary setup was adopted: a 100 μm inner capillary diameter nested within a 600 μm outer tube, operated with an outer-to-inner flow-rate ratio of 1:10 (Fig. S1). After the titration operation, the obtained Au@BTO/ACT microgel spheres were cured under UV light for 1 min, followed by the removal of the oil phase and stirring the microgel spheres in the PDA solution for 30 min. Finally, the precipitates were collected and freeze-dried to obtain SPCM.

To further observe the surface morphology, SPCM were observed by a stereomicroscope. Under the microscope, SPCM exhibited a uniform spherical shape with a diameter of approximately 600 μm (Fig. 2f). In addition, a thin gray layer, representing the PDA coating, was clearly visible on the surface of the gel spheres (Fig. 2g). After freeze-drying, the SPCM's ultrastructure was further scrutinized by SEM. The results showed that SPCM exhibited a typical porous structure (Fig. 2h), with small PDA molecules adhering to the surface (Fig. 2i).

Ultrasound is involved in this research process, and the selection of ultrasound parameters has a significant impact on the experimental results. The ultrasound device provides three levels of output power (0.32 W, 3.2 W, and 6.4 W) and is equipped with an intelligent temperature control protection system to effectively ensure operational safety. Before the experiment, we used an infrared thermometer (Uti260B, Ulide) to monitor the real-time temperature during the in vitro ultrasound irradiation process. The results showed that the probe temperature slowly increased with ultrasound power and duration (Fig. S3a). During the 5-min irradiation time, the maximum temperature difference generated by the probe was ≤1.4 °C (Fig. S3b). The in vitro release rate is an important indicator for evaluating the sustained release effect of composite drug-loaded microspheres. As shown in Fig. S4, ACT can be rapidly released from the SPCM within 7 h under ultrasound, with a cumulative release percentage of approximately 64%, while in conventional release without ultrasound, the cumulative release percentage of ACT is 27% at 11 h. This rapid release allows the nanocomposite to function at the injured site in a relatively short time.

**Fig. 3:**
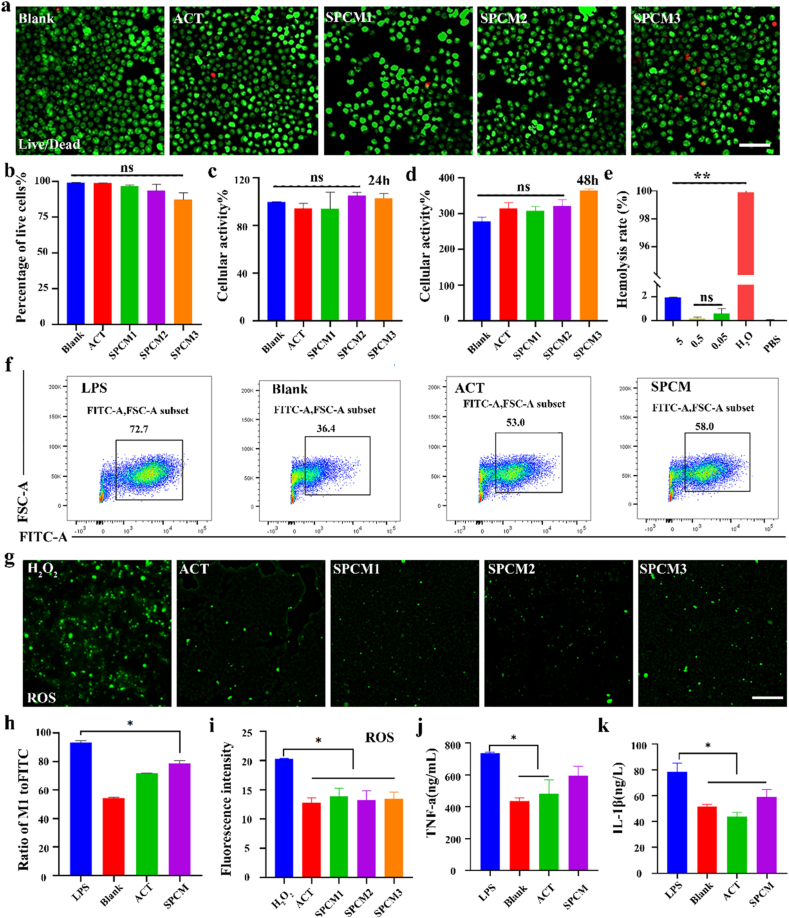
**Characterization of biocompatibility and anti-inflammatory effects of the SPCM. a,** Live/dead cell assay after treatment with ACT and SPCM, live cell (green), dead cell (red), Scale bars, 200 μm. **b,** Quantitative analysis of the percentage of live cells after treatment. **c-d,** CCK-8 assay at 24 h (c) and 48 h (d). **e,** Hemolysis of erythrocytes after co-incubation of SPCM with blood cells and quantitative analysis of hemolysis rate. **f,** FACS analysis for ROS generation in macrophages with exposure to SPCM. **g,** Intracellular ROS in macrophages exposed to ACT and SPCM were visualized by DCFH-DA–derived green fluorescence. Scale bars, 100 μm. **h,** Quantitative analysis of the FACS. **i,** Fluorescence intensity of ROS signal after different treatments. **j-k,** ELISA assay for the secretion of inflammatory factors in cell supernatant with exposure to ACT and SPCM. At least three images were randomly selected from each group for quantification. ∗*P* < 0.05, ∗∗*P* < 0.01 for One Way ANOVA.

SPCM cytocompatibility was examined by exposing RAW 264.7 macrophages to graded doses for 24 h, followed by viability quantification with CCK-8 and fluorescence-based live/dead staining. Fig. 3a shows uniform green Calcein labeling across all SPCM treatments, mirroring the control, with only sporadic PI-positive red nuclei; quantitative comparison revealed no significant inter-group variance in viability (Fig. 3b). The CCK-8 assay results indicated that all groups had high cell viability (Fig. 3c–d), suggesting excellent biocompatibility of SPCM. Additionally, a hemolysis test was conducted to evaluate blood compatibility. The SPCM (5/0.5/0.05 mg/mL) and PBS solutions were clear, in contrast to H_2_O. The hemolysis rates of SPCM groups were below 5%, indicating good blood compatibility (Fig. 3e). In summary, these results demonstrated that SPCM has good biocompatibility, no significant cytotoxicity, and can achieve transformation.

To evaluate the anti-inflammatory and antioxidant capabilities of SPCM, LPS stimulated RAW 264.7 macrophages to produce inflammation. Previous studies have shown that IL-1β and TNF-α, etc are rapidly upregulated in the initial phase of inflammatory damage [33]. In our experiment, macrophages treated with LPS exhibited high levels of IL-1β and TNF-α (Fig. 3j–k). However, After SPCM administration, these expressions were controlled. ROS is a key indicator of oxidative stress levels in endometrial injury [34]. To investigate the antioxidant capacity of SPCM, flow cytometry and fluorescence probe were used to detect intracellular ROS in macrophages stimulated by LPS. As expected, LPS alone provoked a sharp increase in ROS levels relative to untreated controls, while ACT and SPCM reversed this increase, and the two treatment groups were not statistically distinguishable (Fig. 3f and h). Fluorescence images also showed a similar trend, with reduced ROS fluorescence signals after treatment with ACT and SPCM (Fig. 3g and i). The above results suggest that SPCM effectively suppresses the production of inflammatory cytokines and ROS in macrophage cells exposed to LPS. Additionally, the ROS clearance efficiency between each dose group is similar, indicating that SPCM may have a consistent antioxidant effect across the tested concentrations.

The metal nanoparticles in Au@BTO can exert antibacterial activity by interacting with bacterial surfaces, releasing toxic ions to disrupt enzyme function or DNA, dissolving substances, or generating ROS that cause oxidative stress. Therefore, we evaluated the ROS-generating capability of Au@BTO. Terephthalic acid (TA) can capture ·OH to form fluorescent 2-hydroxyterephthalic acid (HTA). By monitoring the fluorescence intensity of HTA at ∼240 nm, we observed the production of ·OH increased in a time-dependent manner with prolonged ultrasound time (Fig. S5a). Moreover, Au@BTO exhibited a higher ·OH generation efficiency than BTO nanocrystals (Fig. S5b). To assess the generation of singlet oxygen (^1^O_2_), 1,3-diphenylisobenzofuran (DPBF) is used as a selective probe, which undergoes degradation when reacting with ^1^O_2_. The fluorescence intensity of DPBF at 426 nm was monitored and found to decrease gradually with US exposure, indicating a time-dependent production of ^1^O_2_ (Fig. S5c). The slope of the ^1^O_2_ generation curve for the Au@BTO + US group was comparable to that of the BTO + US group, but significantly steeper than that of the US group alone (Fig. S5d).

**Fig. 4:**
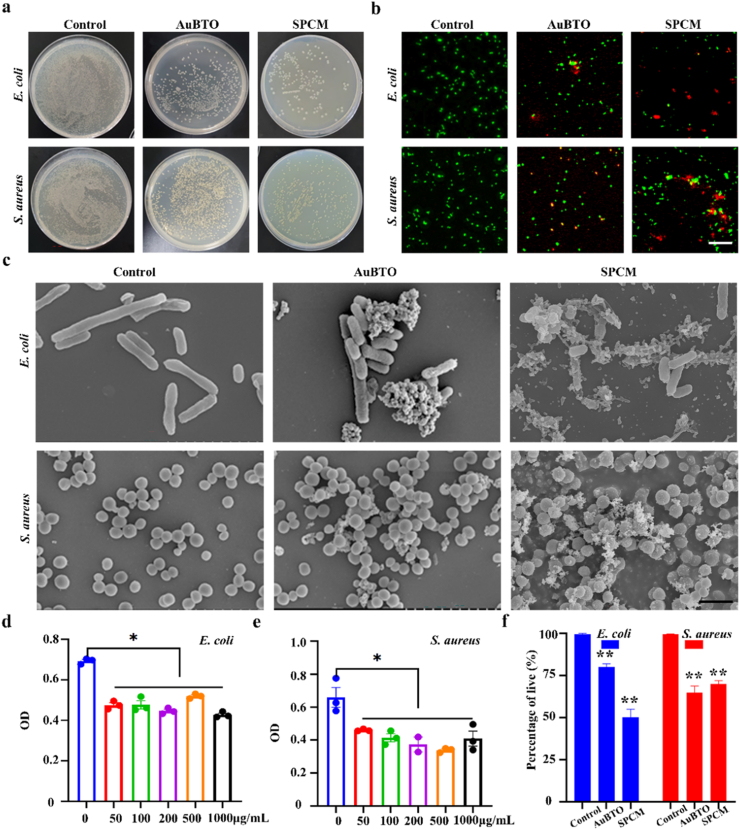
**Characterization of anti-bacterial effects of SPCM. a,** The agar culture plates images of colonies after treatments. **b,** Fluorescence staining of bacterial viability in different groups. Live bacteria (green) and dead bacteria (red). Scale bars, 100 μm. **c,** SEM images of bacteria with different treatments. **d-e,** OD levels of LB culture medium. **f,** Quantitative analysis of bacterial viability. ∗*P* < 0.05, ∗∗*P* < 0.01 for One Way ANOVA.

To study the antibacterial effect of microspheres, *Escherichia coli* (*E. coli*) and *Staphylococcus aureus* (*S. aureus*) serve as representative strains, and detected by counting colony-forming units and visualizing live/dead bacterial staining. Specifically, different concentrations of Au@BTO were mixed with bacterial suspensions and incubated in a sterile shaker at 37 °C for 8 h. Following incubation, measure the absorbance (OD) of each suspension at 570 nm using a microplate reader. The results showed that the OD values of all Au@BTO groups were lower than those of the positive control group, indicating that Au@BTO had antibacterial effects (Fig. 4d–e). Subsequently, three groups of samples were evenly coated on the surface of agar plates. After 24 h of incubation, the number of colonies in the Au@BTO and SPCM groups was significantly reduced compared to control (Fig. 4a). After immunofluorescence staining, control group appeared uniformly green, indicating good bacterial activity. In contrast, the Au@BTO and SPCM groups showed significantly enhanced red signals, indicating effective bacterial killing (Fig. 4b). Further quantitative analysis confirmed that the mortality rates of *E. coli* in the Au@BTO and SPCM groups were 80% and 50%, respectively, while the mortality rate of *S. aureus* in Au@BTO and SPCM groups were 64.67% and 70%, respectively (Fig. 4f). To examine the morphology changes of bacteria, SEM was performed. Untreated bacteria exhibited intact and smooth membranes. In contrast, bacteria treated with Au@BTO and SPCM exhibited severe morphological changes, including wrinkled and distorted cell walls and increased cell membrane permeability (Fig. 4c). In summary, SPCM developed in this study demonstrated significant antibacterial effects.

**Fig. 5:**
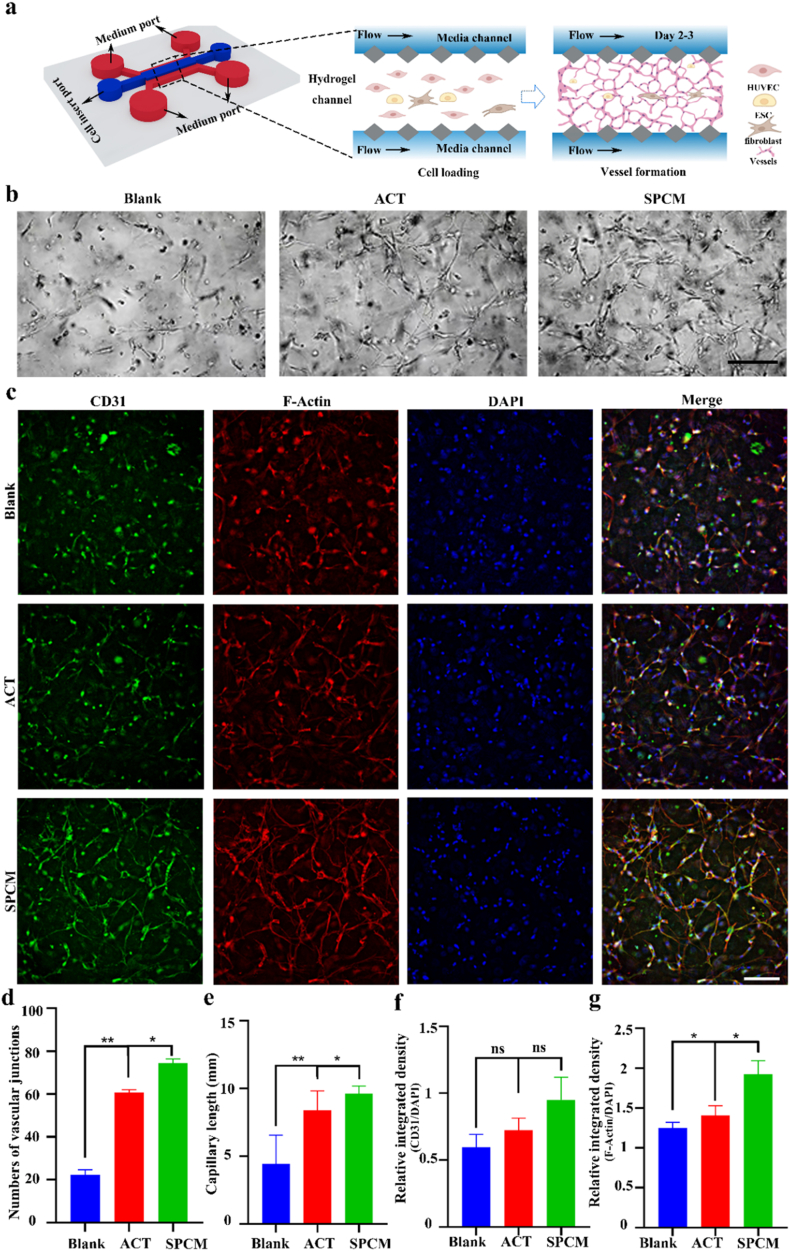
**The impact of ACT and SPCM on tube formation. a,** The model diagram of three-channel microfluidic device and the schematic diagram of the inoculated cells rearranged into interconnected MVN in fibrin hydrogel. **b,** Bright field diagrams of MVN formation in microfluidic devices at different intervention treatments. Scale bars, 100 μm. **c,** Fluorescence MVN images expressing CD31 and F-actin. Scale bars, 100 μm. **d-e,** Quantification of number of vascular junctions and capillary length. **f-g,** Statistical analysis of relative fluorescence signals of CD31 and F-Actin. ∗*P* < 0.05, ∗∗*P* < 0.01 for One Way ANOVA.

Angiogenesis is an important stage in the healing of the endometrium after injury. The application of microfluidic devices in microvascular networks (MVNs) provides a reliable, flexible, and versatile method for microvascular formation under simulated physiological conditions [[35], [36], [37]]. Therefore, we conducted a series of angiogenesis tests based on microfluidic chip to evaluate the angiogenic effects of ACT and SPCM under physiological conditions. In this study, HUVEC and endometrial stromal cells, used as pericytes, were seeded into the central channel in fibrinogen solution (Fig. 5a). Thrombin quickly cross-linked fibrinogen into a stable 3-D gel that entrapped the cells. The two sides of the hydrogel area are medium channels, separated from the gel by square pillars, supplied nutrients and oxygen through trapezoid-post gaps. Inside this scaffold, endothelial cells self-organized into a perfusable microvascular network. As shown in Fig. 5b, over time, the number of vascular junctions and capillaries length under control (blank), ACT and SPCM supplemented gradually increase in the three-channel microfluidic device. The cells reorganized into tubular structures within 3 days and formed interconnected MVNs, with SPCM showing better efficacy than ACT. After 3 days of perfusion culture in the vascular-on-a-chip, immunostaining with CD31 antibody, ZO-1 antibody and F-Actin antibody was applied to evaluate the functionalization of HUVECs. Expression of endothelial-specific markers CD31 and ZO-1 confirmed the tightest intercellular junctions in the SPCM group. F-actin labeling of the cytoskeleton further revealed that this group developed the most highly branched, honeycomb-like microvascular network, followed by ACT (Figs. 5c and S6a). Quantitative analyses of junction number and total vessel length, together with relative fluorescence intensity statistics, all corroborated this hierarchy (Figs. 5d–g and S6c–d). Beside for that, we also conducted tube formation experiment in 96-well plates. It can be seen that HUVECs treated with ACT and SPCM form more tube in the bright field using an optical microscope to visualize cells (Fig. S6b), and further quantitative analysis also confirmed the above results (Fig. S6e–f). These results indicate that SPCM has a pro angiogenic effect through tube formation, and its effect is superior to ACT.

**Fig. 6:**
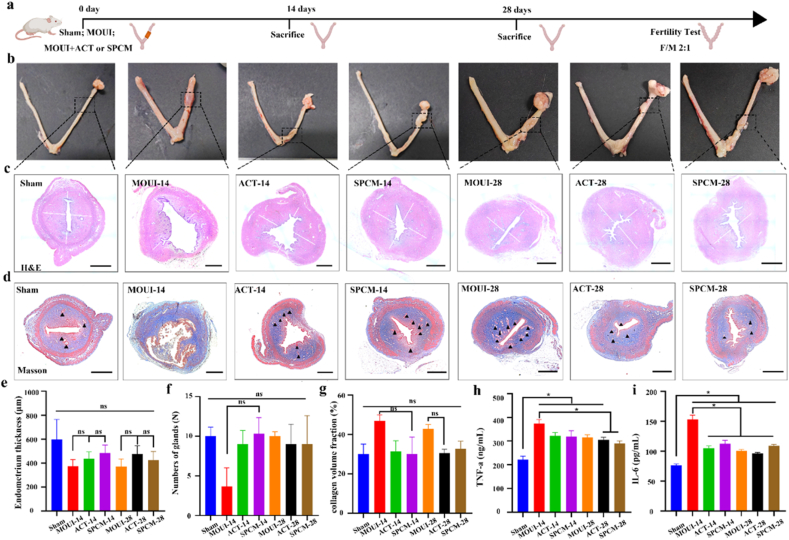
**Therapeutic effects of SPCM in MOUI rat. a,** In-vivo schedule. **b,** Macroscopic uterine appearance. **c,** H&E sections (The white line indicates endometrial thickness). **d,** Masson image (the black triangles indicate glands). Scale bars, 400 μm. **e-g,** Quantitative analysis of endometrial thickness (e), gland counts (f) and collagen volume fraction (g). **h-i,** ELISA assay for the secretion of TNF-α and IL-6 in serum. All the values are represented as the means ± SD (n = 3). ∗*P* < 0.05 for One Way ANOVA.

To further assess the therapeutic efficacy of SPCM on endometrial injury, we established endometrium-injured rats via mechanical damage. The detailed procedure was as follows: initially, rats were anesthetized via intraperitoneal injection of tribromoethanol. The abdomen was opened to expose the right uterine horn. A 0.3-cm-diameter incision was made approximately 0.5 cm from the ovary. Subsequently, the endometrium was completely scraped using a 2.5-mm-diameter micro-curette, with the scraping performed approximately 4 to 6 times. Finally, suture the abdominal incision and disinfect it. In this experiment, rats were randomly divided into four groups: sham group, MOUI group, ACT group, and SPCM group, with 15 rats in each group. On the postoperative days 14 and 28, rats were sacrificed and uterine tissues were collected for histological analysis. On day 28, the remaining female rats were mated with male rats at a ratio of 2:1 to observe fertility restoration (Fig. 6a). The gross morphology of the uterine horns in each group on the days 14 and 28 were presented in Fig. 6b. The control group exhibited a smooth, red, and elastic “Y”-shaped double uterus. On day 14, the damaged area of the uterus in the scraping group appeared as granulation tissue, which was congested, bright red, and soft and moist. By day 28, the damaged area of the uterus had gradually fibrosed to form scar tissue, appearing pale or grayish-white, hard, and tough. To observe the pathological changes of the uterus, we performed H&E staining to examine endometrial morphology, thickness, and gland number. The results showed that with the prolongation of repair time, the endometrium gradually thickened, and the SPCM group had a faster repair rate on day 14 (Fig. 6c). Specifically, the average endometrial thickness of the sham group was 597 μm, with 10 glands; the average thickness of the MOUI-14 group is 372 μm, with only 3 glands; The average thickness of ACT-14 group is 436 μm, with 9 glands; The average thickness of SPCM-14 group is 484 μm, with 10 glands (Fig. 6e–f). Collectively, these data indicate that SPCM promotes repair of damaged endometrium, restores both thickness and glandular density to normal uterine levels. Endometrial fibrosis was gauged by Masson staining, which maps collagen deposition across groups (Fig. 6d). The ratio of blue-stained area to total area reflects the degree of uterine fibrosis. All treated uteri displayed an increase in collagen deposition than sham group on the 14th day after surgery. While the collagen deposition level of SPCM was significantly lower than that of MOUI groups. According to the statistical data 14 days after surgery, collagen occupied 46.84 % of the field in the MOUI group, while ACT group and SPCM group were 31.48% and 30.23%, respectively (Fig. 6g).

**Fig. 7:**
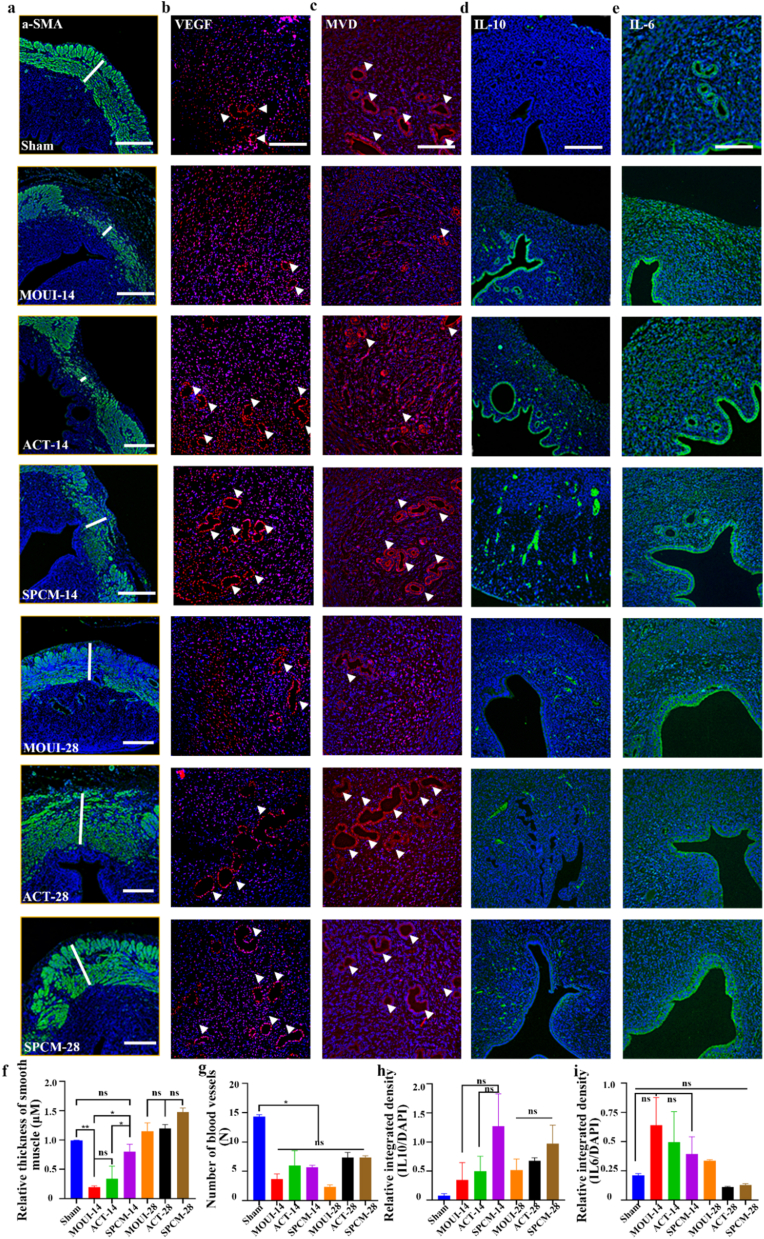
**Characterization of the uterus tissue with SPCM treatment. a,** α-SMA staining gauged smooth-muscle thickness. Scale bars, 100 μm. (The white line indicates smooth muscle thickness). **b-c,** VEGF/MVD immunofluorescence (200 μm) mapped blood vessels. Scale bars, 200 μm. **d-e,** IL-10 and IL-6 staining quantified local inflammation. Scale bars, 200 μm. **g-i,** Quantitative analysis of the molecular expression levels mentioned above in the uterus tissues. ∗*P* < 0.05 for One Way ANOVA.

Myometrium serves as the essential structural foundation of uterine function. It provides the majority of mechanical support for embryonic development during pregnancy and generates contractile force required for fetal delivery at period of pregnancy [38,39]. Alpha-smooth muscle actin (α-SMA) marks mature smooth-muscle, thus available for assess the regeneration of smooth muscle [40]. Immunofluorescence staining results revealed the mechanical damage caused localized loss of the myometrium, leading to reduced α-SMA staining in those areas, yet either ACT or SPCM reinstated it, SPCM accelerating recovery by day 14. (Fig. 7a). Early tissue repair hinges on the rapid establishment of new microvasculature [41]. Vascular endothelial growth factor (VEGF) is the principal regulator of angiogenesis, with its expression levels reflecting the extent of endothelial proliferation, migration, and ultimate capillary assembly [42]. Therefore, we performed immunofluorescence staining of the angiogenesis markers MVD and VEGF. As shown in Fig. 7b–c, MVD and VEGF are typically expressed in sham group, but in the MOUI group, MVD and VEGF-positive vessels were sparse at 14 days and fewer at 28 days, indicating slow repair of the vascular system in the endometrium. Compared with the MOUI group, both ACT and SPCM groups exhibited markedly higher MVD and VEGF levels, suggesting that ACT and SPCM can restore injured endometrium by up-regulating VEGF and MVD, thereby promoting angiogenesis in the damaged endometrium. To evaluate tissue proliferative activity, Ki-67 immunofluorescence staining was additionally performed on paraffin sections collected at 14 and 28 days after transplantation (Fig. S7a). Quantitative analysis revealed a significant time-dependent increase in Ki-67-positive cells, with the SPCM group outperforming the ACT group, which in turn surpassed the MOUI group (Fig. S7b). In summary, these results demonstrate that ACT and SPCM can promote endometrial angiogenesis and myometrial regeneration, with SPCM showing better and faster repair effects.

The inflammatory microenvironment can affect the recovery of endometrial receptivity. When the basal layer is severely damaged, a large number of inflammatory cells quickly accumulate at the site of endometrial injury, triggering an inflammatory cascade reaction and releasing various pro-inflammatory cytokines. Therefore, inhibiting inflammatory factors is key to repairing endometrial injury [43]. To verify the therapeutic effects, we collected blood to detect the content of inflammatory cytokines of IL-6 and TNF-α. The result revealed that pro-inflammatory cytokines were significantly elevated in the MOUI group, while ACT and SPCM treatments brought these mediators sharply down (Fig. 6h–i), indicating that their anti-inflammatory efficacy in injured endometrium. Additionally, immunofluorescence staining results showed that IL-6 was elevated to varying degrees in the modeling group relative to the sham group, but decreased to a certain extent after treatment with ACT and SPCM (Fig. 7e). IL-10 suppresses inflammation by curbing pro-inflammatory cytokine release and modulating the activity of immune cells. In this study, the level of IL-10 was higher in the MOUI, ACT, and SPCM groups compared to the sham group (Fig. 7d). In summary, SPCM can promote endometrial repair by appropriately modulating the intrauterine inflammatory environment.

Biocompatibility testing, as an important means of evaluating the interaction between biomedical materials and tissues, has a wide range of applications and significant importance in the biomedical field. We examined major organs via H&E staining after treatment to assess the biocompatibility of the material. The results showed no obvious structural abnormalities in the heart, liver, spleen, lungs, and kidneys of each group (Fig. S9). These results indicate that the interventions had no significant toxic side effects on major organs and were highly safe. It is worth noting that the Au@BTO used in this study are both non-biodegradable inorganic materials. Although the dosage is extremely low, its transport and metabolic processes in uterine tissue still require attention. The uterus, as an organ with a unique immune microenvironment and periodic remodeling characteristics, has a complex mechanism for clearing substances. Most materials can be excreted with uterine fluid and vaginal secretions, while a small amount may be periodically shed and cleared with the endometrium. Extremely small amounts may be transported and metabolized through the lymphatic system after being engulfed by macrophages. But these pathways are still speculative and require experimental verification. We will further evaluate its long-term safety and explore the feasibility of using biodegradable piezoelectric materials as an alternative solution.

**Fig. 8:**
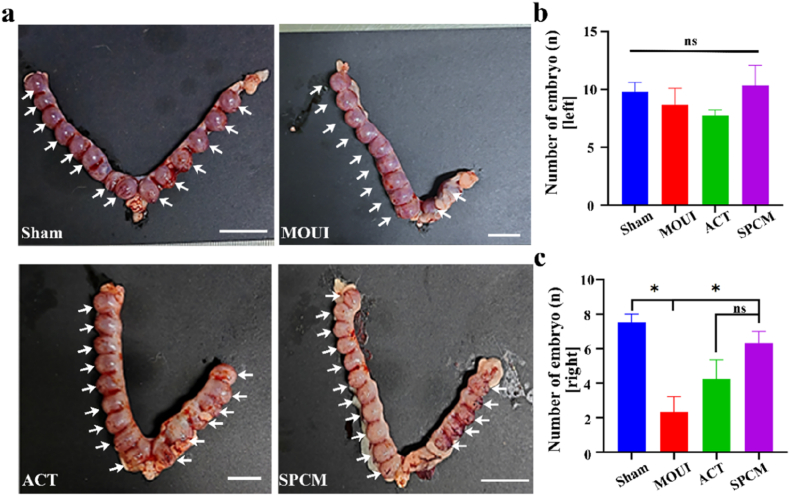
**SPCM-mediated fertility recovery.** a, Uterine horns with embryo (white arrow) in sham group, injured endometrium and injured endometrium with ACT/SPCM. b, Implantation-site counts per horn side (n = 4). ∗*P* < 0.05. Scale bars, 2 cm.

The goal of endometrial regeneration is to restore fertility. In our study, female rats were allowed to mate with male rats after a period of 28 days post-surgery. In order to maintain a consistent ovulation cycle, all female rats were injected with Pregnant Mare Serum Gonadotropin the day before mating to regulate their physiological cycle. On the 10 days after mating, the female rats were euthanized to expose the uterus and evaluate fertility recovery. As shown in Fig. 8a, no embryos were observed in the right uterine horn of the MOUI group, indicating that fertility capability had not been restored in that horn. In contrast, the ACT and SPCM groups both had embryo implantation, with the SPCM group showing the higher number of embryos. To further evaluate the developmental outcomes, we assessed the weight of individual gestational sacs and found no statistically significant difference in weight between successfully implanted gestational sacs (Fig. S8). These results indicate that SPCM was the most effective in restoring fertility in the endometrium following mechanical injury.

## 3 Conclusions

In this study, we integrated multiple advanced principles to design and develop a novel US-responsive core-shell SPCM specifically for endometrial injury repair, with the expectation of significantly improving pregnancy outcomes. The surface-coated PDA possess multiple therapeutic properties, including anti-inflammatory effects, promotion of angiogenesis, and ROS scavenging. The metal nanoparticles in Au@BTO can bind to bacterial surfaces and exert antibacterial activity by releasing toxic ions that disrupt bacterial enzyme functions or DNA. To evaluate the practical value of SPCM, we conducted implantation studies in a rat model of MOUI. The in vivo results indicated that the SPCM could rapidly release, significantly increase endometrial thickness, promote angiogenesis, myometrial regeneration, and effectively alleviate endometrial inflammation by regulating the levels of inflammatory cytokines. Notably, the spatiotemporal microspheres greatly restored the reproductive capacity of rats and significantly enhanced the receptivity of the endometrium. Overall, this study indicates that the novel spatiotemporal piezoelectric microspheres have the potential to become a highly promising choice for future endometrial repair, with unique advantages and broad application prospects.

## 4 Materials and methods

### 4.1 Materials

The cell lines present in this study were obtained from Xiamen Immocell Biotechnology Co., Ltd. Gold (III) chloride trihydrate was bought from Sigma-Aldrich, Co., LLC (St Louis, MO, USA). NH_3_·H_2_O and tert-butyl alcohol were obtained from Aladdin Chemical Co. Ltd. (Shanghai, China). Acteoside (CAS:61276-17-3) was bought from Shanghai Yuanye Bio-Technology Co., Ltd. Lipopolysaccharide (LPS) was bought from Beijing Solarbio Science & Technology Co., Ltd (Beijing, China). α-SMA, MVD, VEGF, IL-6, IL-10 antibodies for rat were manufactured by Proteintech Group, Inc. (Chicago, USA). ELISA kits for the cytokines IL-6 (JM-01597R1) and TNF-α (JM-01587R1) were from Jingmei biotechnology, (Jiangsu, China). Female Sprague‒Dawley rats without Specific pathogens (150-180 g, 6 weeks) were purchased from the Animal Center of Nantong University. All animal experiments were approved by the Animal Management Committee of Nantong University and complied with the ethical review standards (approval number: S20250522-002).

### 4.2 Preparation of SPCM

Material preparation: BTO nanocubes were manufactured by Nanjing XFNANO Materials Tec Corporation. To obtain Au@BTO, 500 mg BTO and 2 mL of NH_3_·H_2_O were mixed and stirred in 100 mL deionized water. Next, 4 mL HAuCl_4_·3H_2_O (10 mg Au^3+^/mL, 99.9 %, Alfa Aesar) was introduced; the mixture was vigorously stirred at 80 °C for 1 h, then washed, dried, and finally calcined at 300 °C for 1 h [24]. To obtain PDA, 0.5 mg/mL Dopamine was dissolved in a 1 M Tris-HCl alkaline solution with pH 8.0 and stirred at 60 °C for 24 h. Manufacturing operation: Firstly, GelMA and ACT are dissolved in deionized water at 60 °C at a mass ratio of 1:1 to obtain a 20 wt% solution. Subsequently, 1% 2-hydroxy-2-methylphenylacetone (HMPP) and 500 μg/mL Au@BTO were added to the solution. Then transfer the above solution to a syringe, and titrate the drug loaded micro gel ball in the microfluidic device. The parameter settings of the microfluidic device are as follows: the inner capillary tube diameter is 100 mm, the outer capillary tube diameter is 600 mm, and the ratio of the outer phase flow rate to the inner phase flow rate is 1:10. After titration, put the obtained micro gel ball under ultraviolet light to cure for 1 min, then remove the oil phase, immerse the micro gel ball in PDA solution, and stir for 30 min. Finally, the precipitates are collected and freeze-dried to obtain SPCM.

### 4.3 Characterization of the SPCM

Stereomicroscopy and SEM (Gemini 300) visualized SPCM morphology, while EDX line- and area-scans mapped elemental distribution.

### 4.4 Hemocompatibility assay

Fresh rat blood was used to evaluate the hemolysis of the samples. Blood samples were obtained from rat. The red blood cells (RBCs) were washed three times and then resuspended in PBS to form a 2.0% suspension of RBC. Then the SPCM (5/0.5/0.05 mg/mL), PBS, and distilled water (1000 μL) was added to the RBC suspension. The tubes were incubated in a 37 °C incubator for 6 h to observe any visible agglutination or hemolysis. 100 μL of the supernatant was transferred to a microplate reader and the absorbance at 450 nm recorded. The hemolysis (%) was then computed with the equation:

% Hemolysis = OD_*Test*_ − OD_*Neg*_/OD_*Pos*_ – OD_*Neg*_

### 4.5 Cytotoxicity assay

The cytotoxicity of the SPCM was assessed using the CCK-8 assay and Calcein-AM/PI double staining. The ACT and SPCM were soaked in the culture medium and incubated at 37 °C. The ACT and the SPCM mixed RAW264.7 macrophages were seeded into a 96-well plate and incubated with each extract (200 mL) for 24 h. After triple PBS rinses, CCK-8 was added for 30 min and absorbance read at 450 nm (Multiskan MK3, Thermo Fisher, USA). Parallel cultures in 24-well plates were stained with Calcein-AM/PI and imaged (Thunder, Leica, USA) to quantify viability.

### 4.6 ROS scavenging

RAW264.7 macrophages were stressed with 0.5 mg/mL LPS for 24 h in the presence of graded doses of ACT or SPCM. Intracellular ROS was then quantified by flow cytometry and visualized by immunofluorescence.

### 4.7 In vitro ROS detection

Prepare a 0.5 mM solution of TA and dissolve it in a 2 mM NaOH solution. Next, add 0.5 mM BTO or Au@BTO Dispersion liquid. After 5 min of US (1.5 W cm^−2^, 1 MHz), the fluorescent spectra of HTA at a wavelength of 240 nm was detected using a UV spectrophotometer. Dissolve 20 μM DPBF with 0.1 mM BTO or Au@BTO, sonicate as above and record DPBF absorption.

### 4.8 Antibacterial activity in vitro

*E. coli* (BNCC133264) and *S. aureus* (ATCC6538) were prepared. The bactericidal effects of Au@BTO and SPCM were evaluated through flat plate coating experiments. Prepare LB agar plate: dissolve 2.5 g of LB culture medium powder and 1.5 g of agar powder in 100 mL ddH_2_O; sterilize the mixture by autoclaving at 121 °C for 15 min; transfer the cooled mixture into disposable sterile culture dishes; store them at 4 °C following solidifications. Take 50 μL of the bacterial solution mentioned above; using a sterile triangular glass rod, evenly spread the bacterial suspension onto the surface of the LB agar plate; place the plates in the incubator and incubate at 37 °C for 24 h.

SEM: after treatment, bacteria were centrifuged (3000–4000 rpm), fixed in 2.5 % glutaraldehyde (3 h), PBS-rinsed, dehydrated through graded ethanol (30–100 %, 15 min each) and twice in tert-butyl alcohol (15 min), freeze-dried, and imaged.

Live/dead assay: pellets were stained with SYTO/PI and viewed (Thunder, Leica,USA).

### 4.9 Tube formation assay

The experimental steps for tube formation assay based on three-channel microfluidic device refer to the method of Wan HY et al., while some content has been adjusted. Specifically, as follows: The vascular chip was purchased from Shenzhen Xirui Biotechnology Co., Ltd. Fibrinogen (20 mg mL^−1^ in PBS, Sigma F8630) and thrombin (7.6 U mL^−1^, Meilunbio MB1368) were mixed 1:1 with HUVECs:HESCs:fibroblasts (2:1:1) to give 10 mg mL^−1^ fibrinogen, 3.8 U mL^−1^ thrombin, 12 × 10^6^ HUVECs mL^−1^ and 6 × 10^6^ mL^−1^ each stromal/fibroblast cell. The blend was loaded into the center channel, gelled 20–30 min (37 °C, 5 % CO_2_). Next, primary endothelial cell culture medium with ACT (10 μg/mL) or SPCM (20 μg/mL) was added into the medium channels. After tube formation, endothelial specific markers CD31, ZO-1 and F-Actin are used for immunofluorescence staining and provide quantitative image analysis.

### 4.10 Construction of a rat model of MOUI

To establish a rat model of MOUI, adult female Sprague-Dawley rats (6 weeks, average weight 150 g) were housed in a temperature-controlled room (20–22 °C) with a 12 h light/dark cycle for one week. During this period, the physiological cycle is regulated to ensure that the estrus cycle of the animal remains consistent before surgery. During the formal surgery, the rats were first anesthetized via intraperitoneal injection of Serazine Hydrochloride (5 mg/kg) and Zoletil (15 mg/kg). All subsequent operations were performed under anesthesia. The surgical procedure involved exposing the right uterine horn. An incision approximately 0.5 cm in length was made at 1/3 point from the bifurcation of the uterus. A micro-curette with a diameter of 2.5 mm was then used to thoroughly scrape the uterine cavity, with the scraping performed about 4-6 times. Finally, the uterine incision was sutured and disinfected to complete the procedure. The animal experiments were approved by the Animal Ethics Committee of Nantong University.

### 4.11 SPCM treatment

60 female rats were randomized into four cohorts of 15: sham (laparotomy only), MOUI (injury, no therapy), ACT (injury + ACT), and SPCM (injury + SPCM). During the construction of the rat model, ACT (100 mg/kg) or SPCM (200 mg/kg) was slowly and evenly transplanted into the wound site at the scratch location. All surgical procedures were performed under conventional anesthesia and sterile conditions. The ultrasound device used in this study is the US PRO 2000™, Model # DU3035. The device is equipped with a temperature protection function: if the treatment head temperature exceeds 107 °F (42 °C), the treatment automatically pauses, indicated by two flashes of the timer light, and will only resume once the temperature drops below 104 °F (40 °C). For the above group, the US irradiation mode used in this study is M-mode, with an output frequency of 1.0 MHz, an output power of 3.2 W, an effective area of 4.0 cm^2^, and output transmitted in pulse form with a 50% duty cycle and an effective intensity of 0.80 W/cm^2^. The irradiation time is 5 min and lasts for 7 days.

### 4.12 H&E and Masson

Uteri harvested on days 14 and 28 were processed for H&E and Masson. Thickness was read as the shortest vertical span between opposing endometrial–myometrial borders. Specific measurement method: Measure the endometrial thickness in four directions of 0°, 90°, 180°, and 270° for each slice, and calculate the average value as the endometrial thickness of this section. The glands were tallied manually. Collagen fraction was extracted from Masson images with ImageJ and expressed as % fibrotic area.

### 4.13 Enzyme-linked immunosorbent assay (ELISA)

Serum was separated and the concentrations of cytokines (IL-6, TNF-α) in the supernatant samples were measured using an ELISA kit (Jingmei, Jiangsu).

### 4.14 Immunofluorescence staining

Immunofluorescence staining was used to detect markers for blood vessels, fibrosis and inflammatory cytokines on days 14 and 28. After dewaxing and rehydration, tissue sections were incubated in 3% H_2_O_2_ and blocking solution, followed by primary antibodies against α-SMA (proteintech, 14395-1-AP, diluted 1:200, China), VEGF (proteintech, 19003-1-AP, diluted 1:200, China), MVD (proteintech, 15331-1-AP, diluted 1:200, China), IL-10 (proteintech, 60269-1-Ig, diluted 1:200, China), IL-6 (proteintech, 26404-1-AP, diluted 1:200, China) and Ki67 (proteintech, 28074-1-AP, diluted 1:400, China) at 4 °C overnight. Secondary incubation and DAPI counter-stain followed. Background-corrected images were captured (Thunder, Leica) and quantified in ImageJ. The mean uterine smooth muscle thickness of damage site was measured in the α-SMA staining (green) cross-section of the uterus. Specific method: The thickness of uterine smooth muscle at the defect site in the incision area was selected for inter group comparison. The sham group showed a complete circular smooth muscle structure. However, on the 14th day after surgery, the uterine smooth muscle did not fully regenerate. There are obvious muscle layer defects on the lumen side (i.e. the injury interface) under the microscope. In addition, the number of blood vessels was recorded in detail according to VEGF and MVD staining.

### 4.15 Statistical analysis

Data analysis and statistics were performed using Prism8.0 software. All quantitative data were tested for normal distribution using the Shapiro–Wilk test. For data that followed a normal distribution, we used parametric tests (one-way ANOVA followed by Tukey's post hoc test for comparisons among multiple groups). For data that did not follow a normal distribution, we applied non-parametric tests (Kruskal–Walli's test followed by Dunn's post hoc test for multiple-group comparisons). *P* < 0.05 was considered statistically significant.

## CRediT authorship contribution statement

**Rui Zhao:** Data curation, Formal analysis, Investigation, Methodology, Writing – original draft. **Shiwen Ni:** Data curation, Investigation, Methodology, Validation. **Xinyu Tao:** Investigation. **Yuxing Liu:** Formal analysis, Investigation. **Nengjie Yang:** Methodology. **Chun Cheng:** Project administration. **Yujuan Zhu:** Conceptualization, Funding acquisition, Project administration, Resources, Writing – review & editing. **Mei Yang:** Conceptualization, Resources, Supervision.

## Funding

This work was also supported by the 2025 Jiangsu Provincial Health International (Regional) Exchange Support Program.

## Declaration of competing interest

The authors declare that they have no known competing interests or personal relationships that could have appeared to influence the work reported in this paper.

## Data Availability

Data will be made available on request.
