# Supplementary material for: Spatiotemporal piezoelectric microspheres for wireless endometrial repair with improved pregnancy outcomes
Source: Mater Today Bio. 2026 Feb 10;37:102915. doi: 10.1016/j.mtbio.2026.102915 (PMC12927068; doi:10.1016/j.mtbio.2026.102915)
Supplement: Multimedia component 1 [file mmc1.docx]

**ssSupporting Information**

**Spatiotemporal piezoelectric microspheres for wireless endometrial repair with improved pregnancy outcomes**

Rui Zhao^1,2 #^, Shiwen Ni^1,#^, Xinyu Tao^3^, Yuxing Liu^3^, Nengjie Yang^3^, Chun Cheng^2^, Yujuan Zhu^1,*^, Yang mei^1,*^

^1^ Research Center of Clinical Medicine, Affiliated Hospital of Nantong University, Medical School of Nantong University, Nantong 226001, China

^2^ School of Medical, Nanjing University of Chinese Medicine, Nanjing, 210023, China

^3^ Research Center of Immunology, Affiliated Hospital of Nantong University, Medical School of Nantong University, Nantong 226001, China

^#^These authors contributing equally to this work.

^*^Corresponding authors: [yujuanzhu@ntu.edu.cn](mailto:yujuanzhu@ntu.edu.cn) (Y. Zhu) and jsyangmei@126.com (M. Yang)

**Methods：**

*1.1 Detection of in vitro release rate*

Mix 10 mg of SPCM with 10 mL of PBS buffer solution and conduct in vitro release experiments at a constant speed of 100 rpm. During the release process, take 500 μL of sample every 2 h, and add an equal volume of PBS to maintain the total volume constant. The extracted sample was filtered through a 0.22 μm microporous filter and the absorbance was measured using a UV spectrophotometer at the maximum absorption wavelength of 372 nm. Calculate the drug concentration in the sample based on the pre-established standard curve, and further calculate the cumulative release rate of the drug.

*1.2 In vitro ROS detection*

Prepare a 0.5mM solution of TA and dissolve it in a 2mM NaOH solution. Next, add 0.5 mM BTO or Au@BTO Dispersion liquid. After 5 minutes of US (1.5 W cm^-2^, 1 MHz), the fluorescent spectra of HTA at a wavelength of 240 nm was detected using a UV spectrophotometer. Dissolve 20 μM DPBF with 0.1 mM BTO or Au@BTO, sonicate as above and record DPBF absorption.

**Figures**

**
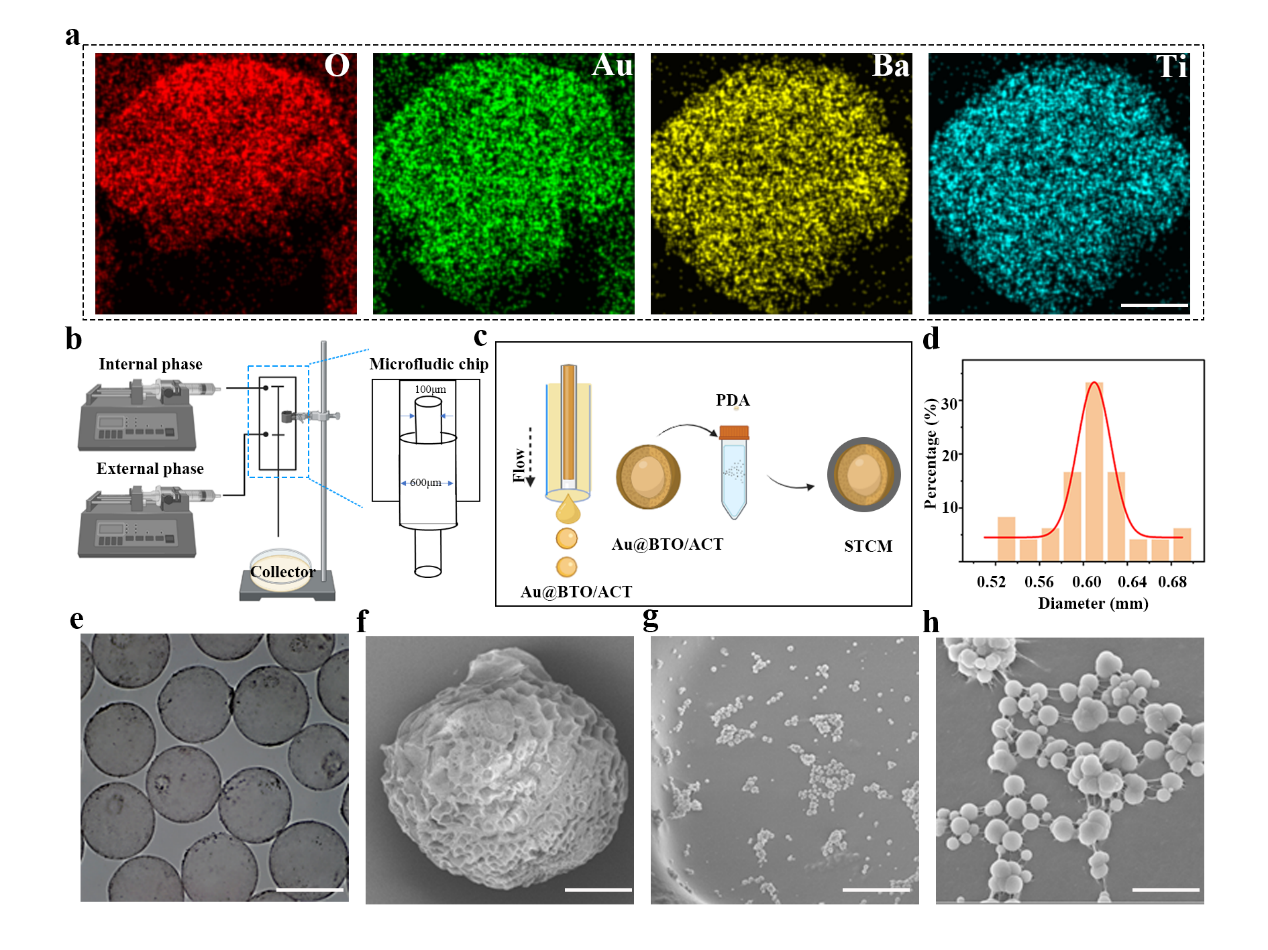
**

**Figure S1.** Device schematic diagram of the microspheres generated by the microfluidic technology.


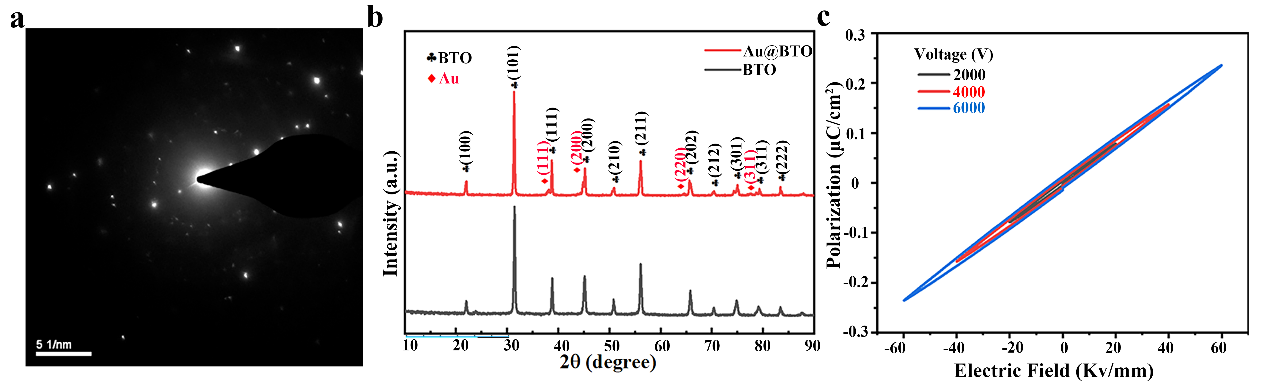
**Figure S2.** **a,** The selected area electron diffraction of the Au@BTO. b, The XRD patterns of the BTO and the Au@BTO. c, The polarization hysteresis loops of Au@BTO under different poling electric fields.


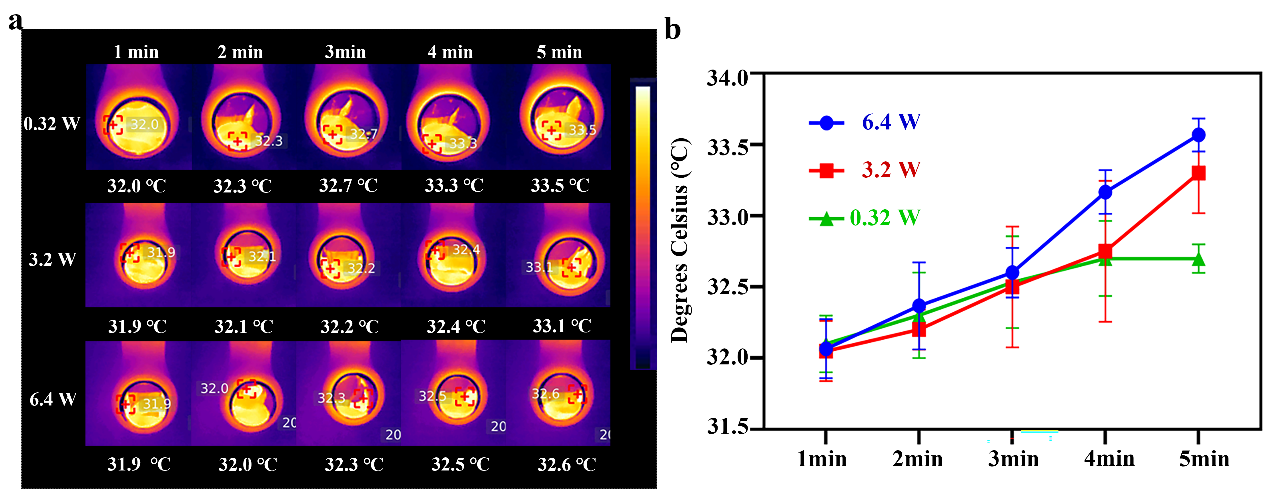


**Figure S3. a,** Near-infrared thermographic images of Ultrasonic probe (5 min). b, Photothermal curves of different modes.


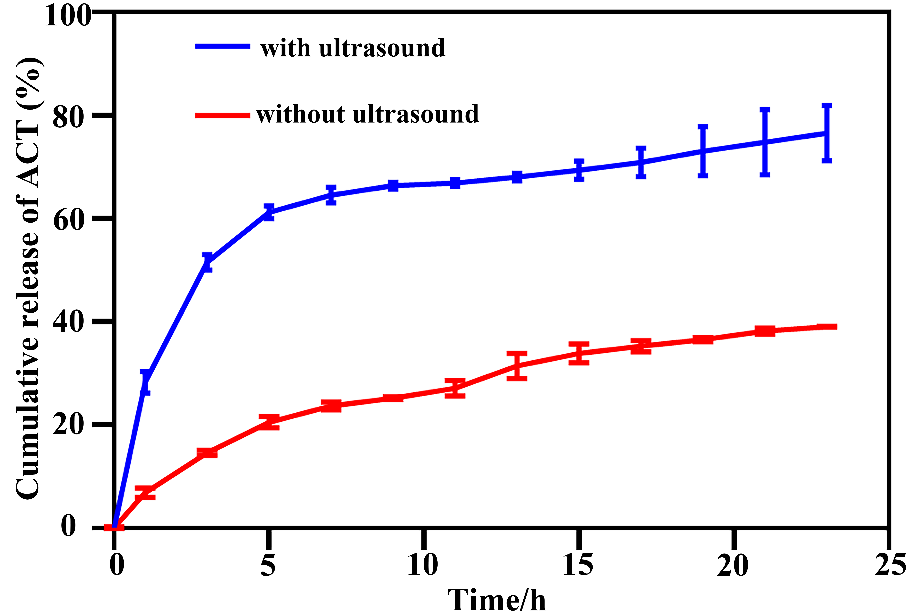


**Figure S4.** Cumulative release of ACT with and without US stimulation (%).


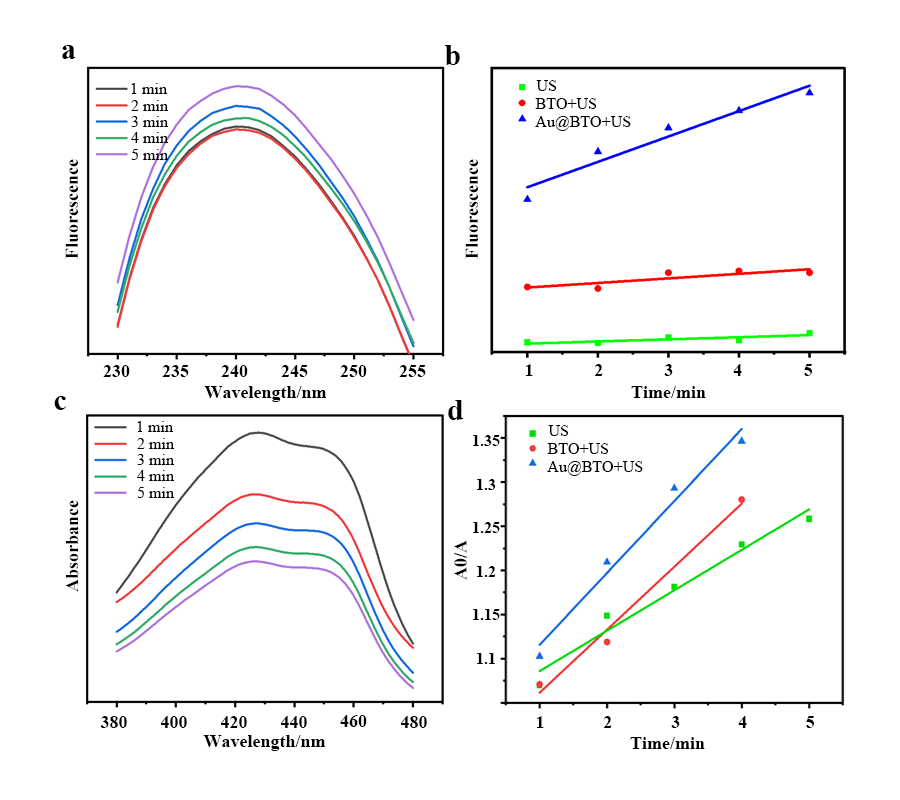
**Figure S5.** **ROS generation for the Au@BTO under US stimuli**. a, Terephthalic acid-derived •OH emission spectra. b, •OH fluorescent intensity at 240 nm. c, DPBF absorption profiles (0–5 min, 0.1 mM Au@BTO + US). d, the rate constant for DPBF decomposition of US, BTO+US and Au@ BTO+US groups under US irradiation for 1–5 min.


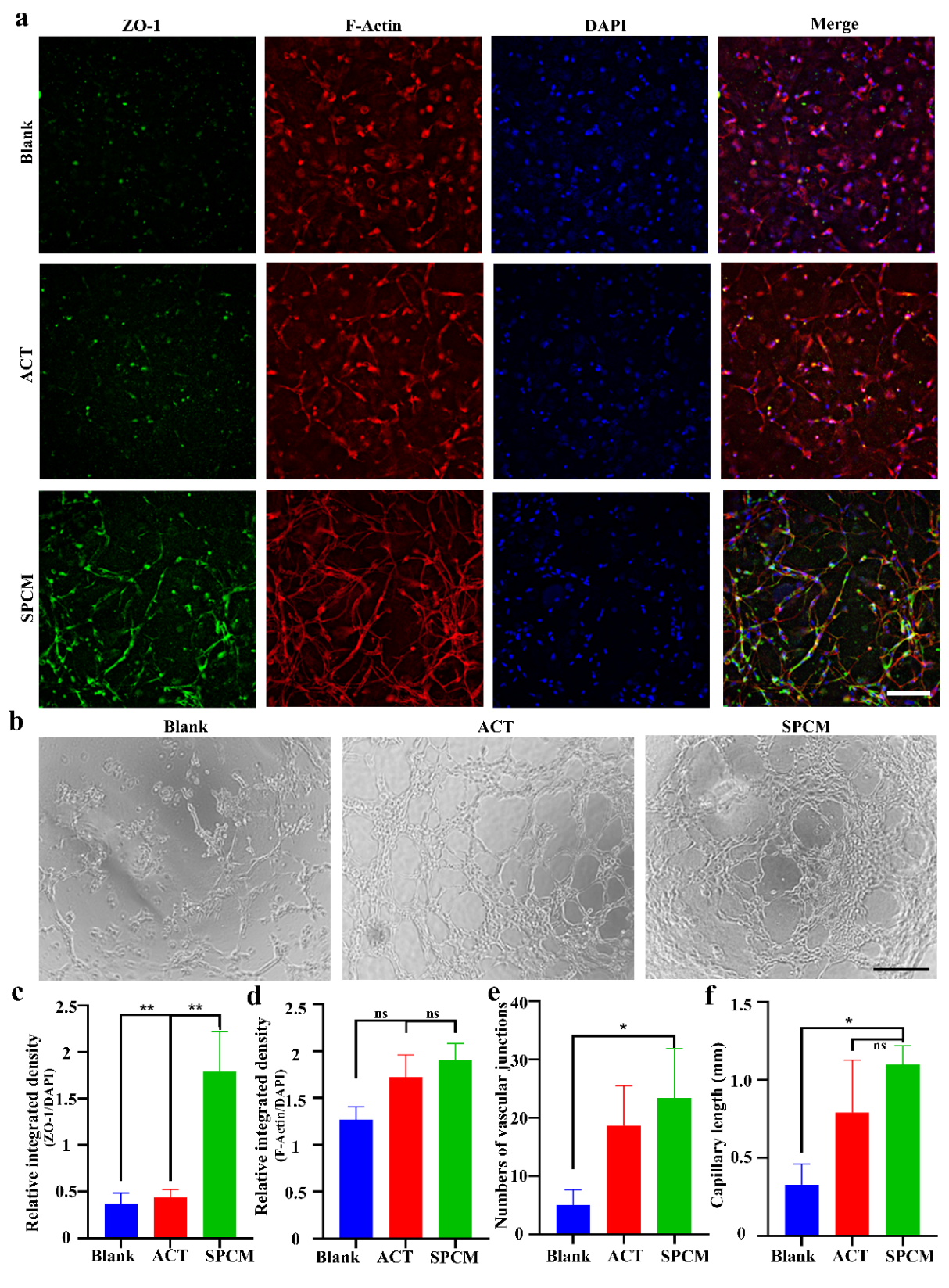
**Figure S6. The impact of ACT and SPCM on tube formation.** a, Fluorescence MVN images expressing ZO-1 and F-Actin. b, Ordinary tube forming experiment. Scale bars, 200 μm. c-d, Statistical analysis of relative fluorescence signals of ZO-1 and F-Actin. e-f, Quantification of number of vascular junctions and capillary length. **P*<0.05, ** *P*<0.01 for One Way ANOVA.


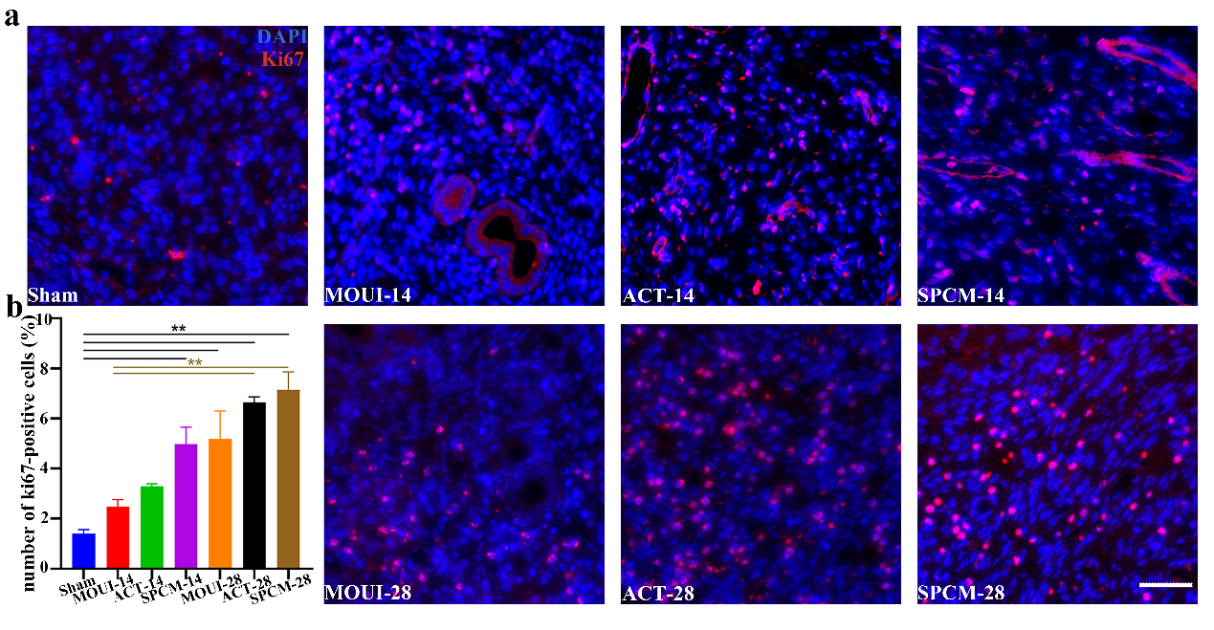
**Figure S7.** a, Uterine sections were co-stained for Ki67 (red) and DAPI (blue). Scale bars, 100 μm. b, Statistical analysis of the number of ki67-positive cells in the uterus tissues from different groups. ** P<0.01 for One Way ANOVA.


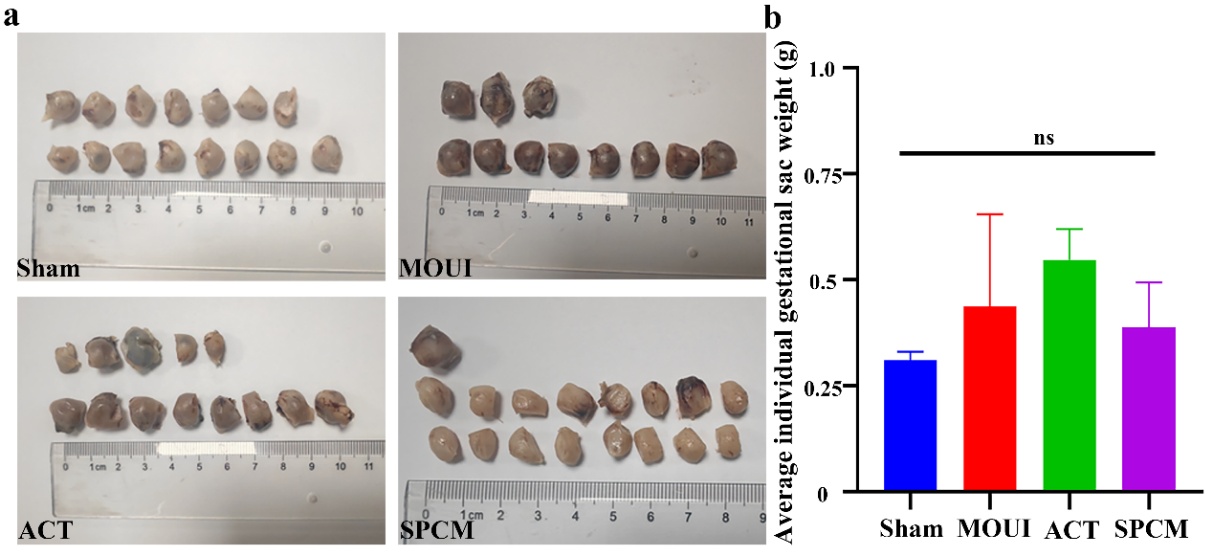


**Figure S8.** a, Single gestational sac distribution map from different groups. b, Average individual gestational sac weight. ns: no significance.


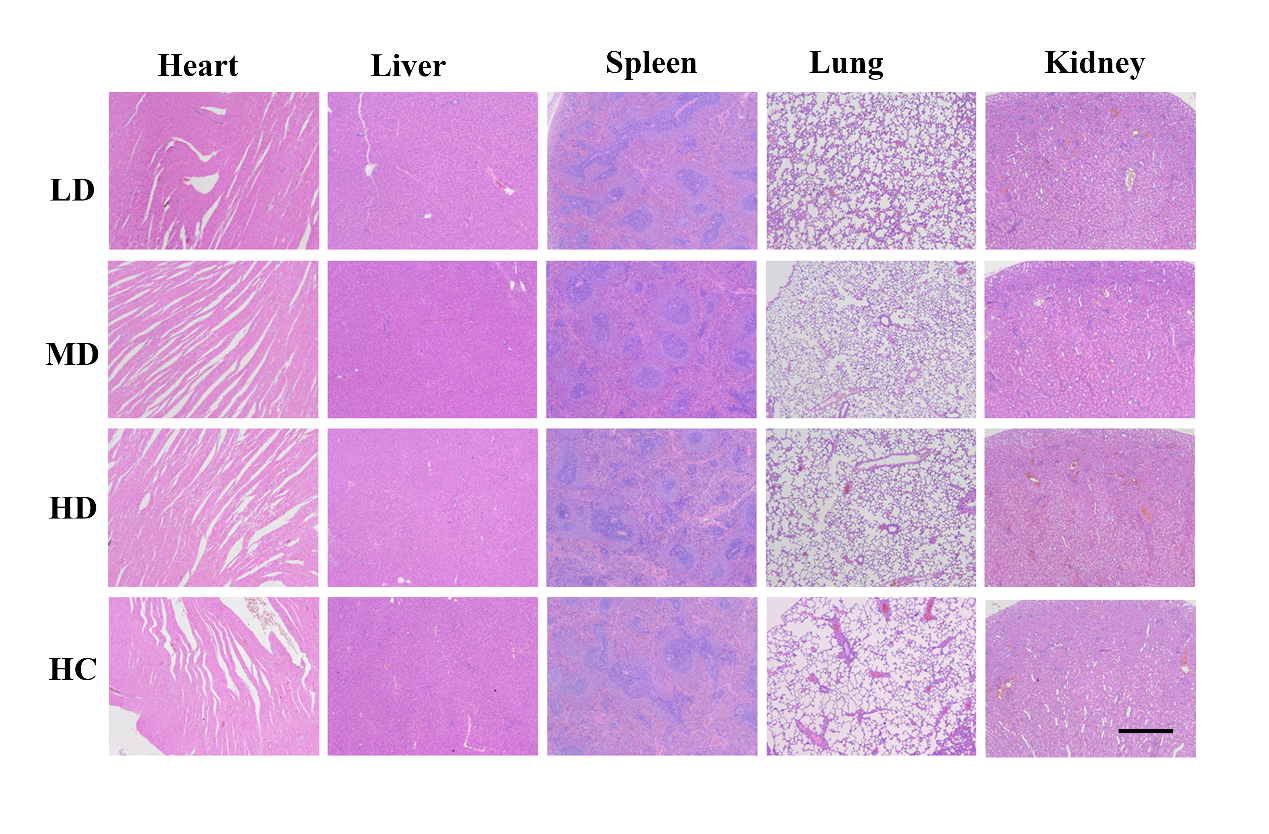
**Figure S9.** H&E staining images of the heart, liver, spleen, lung, and kidney tissue in different groups. Scale bars, 100 μm.
